# Tissue level, activation and cellular localisation of TGF-*β*1 and association with survival in gastric cancer patients

**DOI:** 10.1038/sj.bjc.6603877

**Published:** 2007-07-17

**Authors:** L J A C Hawinkels, H W Verspaget, W van Duijn, J M van der Zon, K Zuidwijk, F J G M Kubben, J H Verheijen, D W Hommes, C B H W Lamers, C F M Sier

**Affiliations:** 1 Department of Gastroenterology and Hepatology, Leiden University Medical Centre, Leiden, The Netherlands; 2 TNO Quality of Life BioSciences, Leiden, The Netherlands

**Keywords:** transforming growth factor-*β*, matrix metalloproteinase, ELISA, fibroblast, tumour microenvironment, immunohistochemistry

## Abstract

Transforming growth factor-*β*1 (TGF-*β*1), a tumour suppressing as well as tumour-promoting cytokine, is stored as an extracellular matrix-bound latent complex. We examined TGF-*β*1 activation and localisation of TGF-*β*1 activity in gastric cancer. Gastric tumours showed increased stromal and epithelial total TGF-*β*1 staining by immunohistochemistry. Active TGF-*β*1 was present in malignant epithelial cells, but most strongly in smooth muscle actin expressing fibroblasts. Normal gastric mucosa from the same patient showed some staining for total, and little for active TGF-*β*1. Active TGF-*β*1 levels were determined by ELISA on tissue homogenates, confirming a strong increase in active TGF-*β*1 in tumours compared to corresponding normal mucosa. Moreover, high tumour TGF-*β*1 activity levels were significantly associated with clinical parameters, including worse survival of the patients. Total and active TGF-*β*1 levels were not correlated, suggesting a specific activation process. Of the different proteases tested, active TGF-*β*1 levels were only correlated with urokinase activity levels. The correlation with urokinase activity suggests a role for plasmin in TGF-*β*1 activation in the tumour microenvironment, resulting in transformation of resident fibroblasts to tumour promoting myofibroblasts. In conclusion we have shown localisation and clinical relevance of TGF-*β*1 activity levels in gastric cancer.

Transforming growth factor-*β* (TGF-*β*) is a multifunctional cytokine, which influences cell differentiation, proliferation, motility and apoptosis (Akhurst and Derynck, 2001; Elliott and Blobe, 2005). Among the TGF-*β* family, which comprises TGF-*β*1, -*β*2 and -*β*3, TGF-*β*1 is most abundantly expressed, especially in various pathological conditions including chronic inflammatory diseases (Marek *et al*, 2002) and cancer (Tsushima *et al*, 1996; Akhurst and Derynck, 2001; Elliott and Blobe, 2005; Bierie and Moses, 2006). Transforming growth factor-*β*1 has been shown to reduce the immune response (Beck *et al*, 2001), stimulate angiogenesis (Choi *et al*, 1997; Derynck *et al*, 2001; Bertolino *et al*, 2005), increase synthesis of proteolytic enzymes (Seomun *et al*, 2001; Kim *et al*, 2004) and stimulate extra cellular matrix (ECM) deposition (Cheng and Lovett, 2003) in the tumour microenvironment.

Several studies examined the role of TGF-*β*1 in gastric cancer. Studies on TGF-*β*1 mRNA showed expression in both normal and tumour tissue, but levels in gastric tumours were strongly upregulated (Naef *et al*, 1997; Park *et al*, 2002). Immunohistochemical studies showed TGF-*β* expression in tumour cells (Naef *et al*, 1997; Park *et al*, 1997; Maehara *et al*, 1999; Saito *et al*, 1999) and sporadic in fibroblasts (Naef *et al*, 1997). Positive TGF-*β*1 immunostaining was found to be related to invasion and metastasis of gastric cancer (Nakamura *et al*, 1998; Maehara *et al*, 1999). Finally, gastric cancer patients showed strongly increased tissue TGF-*β*1 levels and, unexpectedly, reduced serum TGF-*β*1 levels (Park *et al*, 1997).

Transforming growth factor-*β*1 is synthesised as an inactive precursor, the large latent complex consisting of a TGF-*β* dimer, the latency-associated protein (LAP) and latent TGF-*β* binding protein (LTBP) for localisation and binding to the ECM (Mazzieri *et al*, 2005). Before TGF-*β*1 can exert its biological effects, LAP and LTBP have to be dissociated. This can occur by conformational changes (Murphy-Ullrich and Poczatek, 2000; Annes *et al*, 2003; Asano *et al*, 2006), proteolytic cleavage (Lyons *et al*, 1990; Taipale *et al*, 1992; Dallas *et al*, 2002; Wang *et al*, 2006), irradiation (Barcellos-Hoff *et al*, 1994; Ehrhart *et al*, 1997) or by an acid environment (Jullien *et al*, 1989). The complex release mechanism of TGF-*β*1 might implicate that high total TGF-*β*1 has no biological consequences without the presence of appropriate activation mechanisms in the tumour microenvironment. Therefore, measuring TGF-*β*1 activity levels and the localisation in cancer could be more informative regarding the state of cancer progression.

We studied cellular localisation and levels of active TGF-*β*1 and the activation process in gastric cancer. We used an ELISA, which specifically detects active TGF-*β*1, to examine endogenously active as well as total (acid-activated) TGF-*β*1 levels in gastric cancer tissue and showed cellular localisation of active TGF-*β*1 by immunohistochemical staining. To study the activation process, tissue levels of several proteases, putatively involved in the activation process, were determined and analysed for correlations with TGF-*β*1 activity levels.

## MATERIALS AND METHODS

### Patient population

Fresh tissue specimens from a total of 51 patients (34 ♂, 17 ♀) undergoing resection for primary gastric adenocarcinoma at the department of Oncologic Surgery, Leiden University Medical Centre, were collected as described before (Kubben *et al*, 2006). Patient characteristics and clinicopathological parameters are shown in Table 1. Tissue specimens were homogenised in Tris/Tween-80 buffer, and protein concentrations were determined as described previously (Sier *et al*, 1996).

### ELISA for total and active TGF-*β*1

The levels of total and active TGF-*β* were measured using a human TGF-*β*1 duo-set (DY240) with a substrate reagent pack (DY999) according to the manufacturers' instructions (both R&D Systems Europe, Abingdon, UK). This ELISA specifically detects active TGF-*β*1 and does not cross-react with other subtypes of TGF-*β*. Total TGF-*β*1 levels were determined by acid activation (1 M hydrochloric acid, 30 min, room temperature) of the latent TGF-*β*1 in the homogenate. Untreated and activated (30 *μ*l) samples from the same homogenate were assayed in parallel.

### Assays for urokinase, urokinase receptor, plasminogen activator inhibitors, matrix metalloproteinases and tissue inhibitors of matrix metalloproteinases

Total antigen levels of urokinase plasminogen activator (uPA), plasminogen activator inhibitors (PAI-1 and -2), urokinase plasminogen activator receptor, matrix metalloproteinases (MMP-2, -7, -8, -9) and tissue inhibitors of matrix metalloproteinases (TIMP-1 and -2) were determined using previously described ELISAs (Ganesh *et al*, 1994; Sier *et al*, 1994; Hanemaaijer *et al*, 1998; Kubben *et al*, 2006). The bioactivity assays for uPA, MMP-2 and MMP-9 were performed as described before (Hanemaaijer *et al*, 1998; Sier *et al*, 2000; Kubben *et al*, 2006).

### Immunohistochemistry

Tissue samples from the same tumours as used for homogenates were formalin fixed, dehydrated and embedded in paraffin. For cryosections, unfixed tissue was embedded in OCT (Sakura Finetek Europe BV, Zoeterwoude, The Netherlands) and snap-frozen in liquid nitrogen. Paraffin sections (5 *μ*m) were deparaffinised, blocked in 0.3% hydrogen peroxide (H_2_O_2_) in methanol for 20 min and rehydrated through graded alcohol. Antigen retrieval was performed by boiling in a 0.01 M citrate solution (pH 6.0) for 10 min in a microwave oven. After being rinsed in phosphate-buffered saline (PBS), the sections were incubated with the primary antibodies (in PBS/1% bovine serum albumin (BSA)): mouse anti-pan-cytokeratin (1 : 1000, clone C11, Santa Cruz Biotechnologies, Santa Cruz, CA, USA), mouse anti-vimentin (1 : 400, clone V9, Santa Cruz Biotechnologies), mouse anti-smooth muscle actin (anti-SMA; 1 : 1000, clone ASM-1, Progen, Heidelberg, Germany), mouse anti-TGF-*β*1 (1 : 1000, Anogen, Mississauga, Ontario, Canada) or rabbit anti-phospho-smad-2 (p-smad-2; 1 : 1000, kindly provided by Professor/Dr P ten Dijke; Persson *et al*, 1998) overnight at room temperature. After washing, the sections were incubated with biotinylated goat anti-mouse (1 : 200) or goat anti-rabbit (1 : 400, both Dako, Glostrup, Denmark) secondary antibodies (in PBS/1% BSA) for 30 min, followed by washing and incubation with streptavidin–avidin–biotin complex/HRP (Dako) for 30 min. The brown colour was developed by 0.004% H_2_O_2_ (Merck, Darmstadt, Germany) and 0.05% diaminobenzidine tetrahydrochloride (Sigma, Schnelldorf, Germany) in 0.01 M Tris–HCl pH 7.6 for 10 min. The slides were counterstained with Mayer's haematoxylin (Merck) except for the p-smad-2 staining, which were shortly counterstained with methyl green, diluted in 0.1 M sodium acetate buffer pH 4.2. Sections were dehydrated and mounted in entellan (Merck).

Frozen sections (4 *μ*m) were fixated in ice-cold acetone (10 min), washed with PBS and incubated overnight (4°C) with the primary antibodies described below: anti-pan-cytokeratin (1 : 16000), anti-vimentin (1 : 800), mouse anti-SMA (1 : 2000), rabbit anti-active TGF-*β*1 (1 : 800, Promega, Madison, WI, USA) or phospho-smad-2 antibody (1 : 800). Further, the sections were treated as described above and were counterstained with Mayer's haematoxylin. Negative control sections were included by omitting the primary antibodies.

Photomicrographs of representative sections were taken with a Leica DMLB microscope equipped with a Leica DC500 camera.

### Statistical analysis

Differences between normal and tumour values were calculated using the Mann–Whitney *U*-test. For survival analyses, the clinicopathological parameters were dichotomised as described before (Sier *et al*, 1996). Cutoff values for TGF-*β*1 were optimised using ROC analyses. Multivariate survival analyses were performed with the Cox proportional hazards method by separately adding variables to the dichotomised clinicopathological parameters. Survival curves were constructed using the method of Kaplan and Meier including log-rank tests. Differences were considered significant when *P*⩽0.05. The analyses were performed using the SPSS statistical package (Release 12.01, SPSS Inc., Chicago, IL, USA).

## RESULTS

### Tissue TGF-*β*1 levels and clinicopathological characteristics

**Figure 1:**
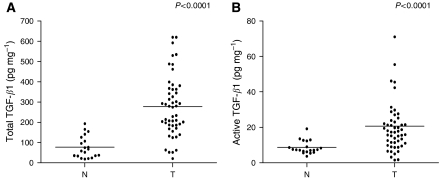
Total and active TGF-*β*1 levels in tumour and corresponding normal tissue homogenates. Total (**A**) and active (**B**) TGF-*β*1 levels are significantly upregulated in tumours (T) compared to corresponding normal (N) tissue (both *P*<0.0001).

Active TGF-*β*1 was detectable in all 51 tumour homogenates with concentrations of 1.6–81.3 pg mg^−1^ protein. Total TGF-*β*1 levels ranged from 21.1 to 620.1 pg mg^−1^ protein. Active and total TGF-*β*1 levels were significantly (*P*<0.0001) increased in gastric cancer tissue compared with adjacent normal tissues (*n*=20; Figure 1A and B). ROC analyses revealed that total as well as active TGF-*β*1 were good diagnostic discriminators between normal and tumour tissue with AUC values of, respectively, 0.91 (*P*=0.03) and 0.82 (*P*=0.05). Tumour levels of active TGF-*β*1 did not correlate significantly with total levels (*ρ*=0.255; *P*=0.071, *n*=51), indicating that the amount of active form is not dependent on the total TGF-*β*1 pool present. The correlation of TGF-*β*1 levels with clinicopathological parameters is presented in Table 1. Active TGF-*β*1 levels were significantly increased in tumours localised in the cardia, in tumours with invasion limited to the subserosa and in tumours with a high inflammation grade. Total TGF-*β*1 levels were enhanced in tumours with high tumour node metastasis (TNM) classification or large diameter.

### Cellular localisation of active and total TGF-*β*1 in gastric cancer

**Figure 2:**
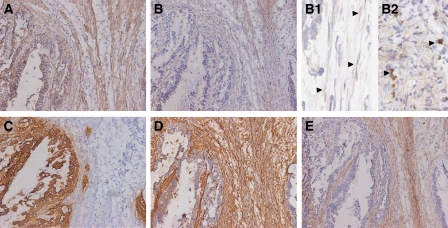
Cellular localisation of active TGF-*β*1 in gastric cancer. Immunohistochemical staining of gastric carcinomas (sequential frozen sections). Staining pattern for active TGF-*β*1 (**A**) corresponds to phospho-smad-2 staining (**B**). Inserts B1 and B2 show nuclear localisation (arrowheads) of p-smad-2 in the myofibroblasts and malignant cells, respectively. As shown by staining for pan-cytokeratin (**C**, epithelial marker), vimentin (Vim, **D**, mesenchymal marker) and SMA (**E**, smooth muscle/myofibroblast marker), TGF-*β*1 activity is observed in malignant cells and in Vim+/SMA+ cells (myofibroblasts). Magnification × 200, -B1–B2 × 630.

To determine the cellular localisation of active TGF-*β*1 in gastric cancer, we stained frozen sections for active TGF-*β*1 (Figure 2A). To confirm the staining being representative for TGF-*β*1 activity, sections were also stained for phosphorylated smad-2 (p-smad-2), indicating TGF-*β* signalling and therefore the presence of active TGF-*β* (Massague *et al*, 2005; Blaney Davidson *et al*, 2006) (Figure 2B). Nuclear localisation of p-smad-2 in myofibroblasts and in malignant cells is shown in Figure 2 panels B1 and B2, respectively.

**Figure 3:**
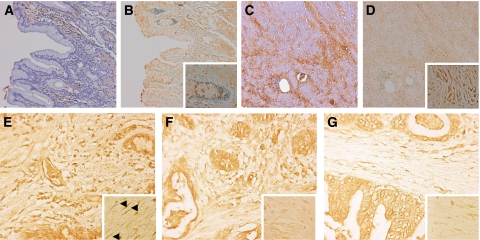
Total TGF-*β* and p-smad-2 staining on paraffin-embedded gastric cancer tissue sections. Staining on normal gastric mucosa shows some staining for total TGF-*β* (**A**) and almost no staining for p-smad-2 (**B**, insert × 400). Both are strongly increased in corresponding tumour tissue (**C**, total TGF-*β*1; **D**, p-smad-2, insert × 400). (**E**–**G**) p-smad 2 staining in three different gastric carcinomas with high (81.3 pg mg^−1^, **E**), median (21.1 pg mg^−1^, **F**) and low active TGF-*β*1 levels (1.6 pg mg^−1^, **G**). A strong decrease in nuclear staining (inserts **E**–**G**, magnification × 630, arrowheads indicate intense nuclear staining in myofibroblasts in **E**) is observed especially in myofibroblasts (staining for pan-cytokeratin, vimentin and SMA on sequential sections, not shown). Magnification × 200.

Staining for both TGF-*β*1 and p-smad-2 was most pronounced in vimentin-positive (Figure 2D) and SMA-positive (Figure 2E) myofibroblasts and in pan-cytokeratin-positive malignant cells (Figure 2C). As, in our hands, staining for total TGF-*β*1 was not detectable on frozen sections from gastric cancer specimens, we stained 10 paraffin-embedded tissue sections from the above-described patient group. Tumours showed strongly increased epithelial and stromal staining for total TGF-*β*1 (Figure 3C) compared to normal gastric mucosa (Figure 3A). Active TGF-*β*1, represented by p-smad-2 staining (Figure 3D, staining for active TGF-*β*1 was not applicable to paraffin sections), was increased in both epithelial as well as stromal cells compared to little staining in normal tissue (Figure 3B).

Figure 3E–G illustrates the association between active TGF-*β*1 ELISA data and the p-smad-2 immunohistochemical staining, indicating TGF-*β* activity. Three carcinomas with decreasing active TGF-*β*1 tissue levels (high, median and low) were stained for p-smad-2 showing decreased nuclear staining for p-smad-2 in the malignant cells and even stronger in the SMA-positive myofibroblasts (SMA, vimentin and pan-cytokeratin staining on sequential sections, not shown).

### Prognostic relevance tissue TGF-*β*1 levels

**Figure 4:**
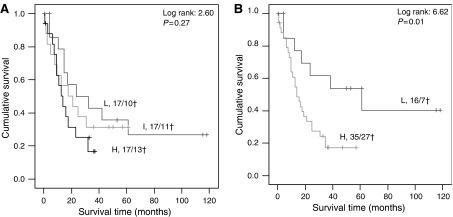
Kaplan–Meier survival analysis for tumour TGF-*β*1 tissue levels. (**A**) Kaplan–Meier analysis showed a stepwise decrease in survival for patients divided in tertiles based on active TGF-*β*1 levels. L=low (<12.56 pg TGF-*β*1 mg^−1^ protein), I=intermediate (12.56–21.28 TGF-*β*1 mg^−1^ protein), H=high (>21.28 TGF-*β*1 mg^−1^ protein), x/y†=number of patients/number of patients deceased. (**B**) Kaplan–Meier analysis showed a significant shorter survival for patients with either high active or high total TGF-*β*1 tissue levels. L=low (active TGF-*β*1<15 pg TGF-*β*1 mg^−1^ protein or total TGF-*β* <400 pg ml^−1^) and H=high (active TGF-*β*1>15 pg TGF-*β*1 mg^−1^ protein or total TGF-*β* >400 pg ml^−1^).

Kaplan–Meier survival curves, using tertiles (cutoff values <12.56; 12.56–21.28; >21.28 pg TGF-*β*1 mg^−1^ protein; Figure 4A) or quartiles (not shown), showed a stepwise correlation for active and total TGF-*β*1 levels with tumour-associated survival. Because of the small group size, the patients were dichotomised for active and total TGF-*β*1 using ROC-based optimal cutoff values (15 (active) and 400 (total) pg TGF-*β*1 mg^−1^ protein, respectively) for further analyses. High tumour levels of active and total TGF-*β*1 were significantly correlated with worse survival (log rank 4.88, *P*=0.027 and log rank 3.96, *P*=0.047, respectively). Figure 4B shows a Kaplan–Meier curve, presenting a combination of either high total or high active TGF-*β*1, which resulted in a higher significance value, confirming the independence of both parameters. To evaluate the validity of the chosen TGF-*β*1 cutoff values, we used the same cutoff values again for the group including 29 more recently collected gastric cancer patients, where active TGF-*β*1 kept its prognostic significance and the hazard ratio increased (*n*=80, HR 6.09, *P*=0.014). The prognostic value of TGF-*β*1 was further evaluated using Cox proportional hazard analyses (Table 2). Particularly active TGF-*β*1 and the combined TGF-*β*1 levels were statistically significantly correlated with survival. In multivariate analysis with the clinicopathological parameters (Table 2), the combined TGF-*β*1 level remained statistically significant in multivariate tests.

### Proteolytic TGF-*β*1 activation

Because TGF-*β*1 is at least partly activated by proteolytic cleavage, we evaluated the total and active TGF-*β*1 levels for correlations with likely candidate proteinases involved in TGF-*β*1 activation and with PAI-1, a presumed secondary marker of TGF-*β*1 activity, in the gastric cancer homogenates (Table 3). Total TGF-*β*1 levels showed significant correlation only with total MMP-2 antigen levels, whereas active TGF-*β*1 levels only showed statistical significant correlation with urokinase (uPA) activity, not with total uPA protein levels.

## DISCUSSION

Numerous studies have shown the involvement of TGF-*β*1 in different types of cancer, including gastric, colorectal and breast cancer (Naef *et al*, 1997; Nakamura *et al*, 1998; Maehara *et al*, 1999; Saito *et al*, 1999; Desruisseau *et al*, 2006). Most of these studies assessed tissue TGF-*β*1 levels by mRNA expression, immunohistochemistry or serum TGF-*β*1 levels, which give either less information on the actual protein levels, are difficult to quantify or do not reflect local effects. A recent study on TGF-*β*1 levels in breast cancer tissue homogenates showed a significant relation of increased tissue total TGF-*β*1 levels with disease-free survival (Desruisseau *et al*, 2006). In the present study we observed a similar relation of tissue total TGF-*β*1 level with survival of gastric cancer patients. Although upregulation of TGF-*β*1 is common in various types of cancer, it is not commonly regarded as a prognostic factor for survival. This is probably due to the fact that the release of the biologically active TGF-*β*1 from the latent complex is crucial for the involvement of TGF-*β*1 in pathological processes. Active TGF-*β*1 levels have hardly been studied because of the absence of sensitive assays, which specifically detect active TGF-*β*1 in the low concentrations observed *in vivo*. We optimised an existing ELISA and were able to detect endogenous TGF-*β*1 levels (without acid activation) in all gastric cancer homogenates. Furthermore, the localisation of active TGF-*β*1 in these cancers was shown by immunohistochemical staining for active TGF-*β*1 and its signalling molecule p-smad-2. In a sequential series of tumours with decreasing active TGF-*β*1 levels (ELISA), we also observed strongly decreasing nuclear staining pattern in the myofibroblasts. Active TGF-*β*1 levels showed association with localisation, invasion, inflammation and survival of gastric cancer patients. As expected, the association of active TGF-*β*1 levels with survival was indeed stronger compared to total TGF-*β*1 level. There was no correlation between active and total TGF-*β*1 levels, implying that the activation was not dependent of the total pool latent TGF-*β*1 present in the tumour microenvironment. As a consequence, total TGF-*β*1 levels showed association with other parameters, that is tumour diameter and TNM stage, whereas there was no association of these parameters with active TGF-*β*1 levels.

Localisation of active TGF-*β*1 is observed in inflammatory- and tumour cells and especially in tumour-associated myofibroblasts, implying that increased levels of activated TGF-*β*1, more than overall TGF-*β*1 levels, are associated with accumulation of myofibroblasts in gastric cancer. Indeed, active TGF-*β*1 can induce transdifferentiation from fibroblasts to myofibroblasts in the tumour microenvironment, which show an increased expression of various proteolytic enzymes including MMPs (Dwivedi *et al*, 2006). In turn, these proteases, including plasmin (George *et al*, 2005), MMP-2 (Wang *et al*, 2006), MMP-3 (Maeda *et al*, 2002) and MMP-13 (D'Angelo *et al*, 2001), have been shown to be involved in TGF-*β*1 activation. In our study we observed a significant relation between active TGF-*β*1 levels and urokinase activity, implying plasmin, via urokinase-mediated plasminogen activation, as a principal candidate of latent TGF-*β*1 activation. The investigated MMPs showed no significant correlations with active TGF-*β*1 levels. For TIMP-1 and -2, we observed a weak negative correlation with TGF-*β*1 activity, also observed *in vivo* in a recent study (Dasgupta *et al*, 2006). Total TGF-*β*1 levels only correlated significantly with MMP-2 levels in homogenates. Immunohistochemistry showed TGF-*β*1 activity present in different cell types, probably with different activators in the tumour microenvironment. This explains why in a homogenate it is unlikely that a strong significant association with one specific MMP will be observed.

In conclusion, we have shown that total and active TGF-*β*1 levels are increased in gastric tumour tissue and that both are of prognostic relevance in gastric cancer. Active tissue TGF-*β*1 levels showed association with clinicopathological parameters and with uPA activity, indicating a possible role for plasmin in TGF-*β*1 activation in gastric cancer. Immunohistochemical studies showed strong expression of active TGF-*β*1 in the myofibroblast population. We propose that increased proteinase activity in the tumour microenvironment leads to increased ECM-bound latent TGF-*β*1 activation, resulting in transformation of resident fibroblasts to tumour promoting myofibroblasts. Further studies on a larger group of patients should be performed to establish the prognostic value of active tissue TGF-*β*1 levels in gastric cancer and further elucidate the mechanism of latent TGF-*β*1 activation.

## Figures and Tables

**Table 1:** Median levels^a^ of total and active TGF-*β*1 in gastric carcinomas in relation to clinicopathological parameters

		**Active TGF-*β*1**			**Total TGF-*β*1**		
	***n***	**Median**	**Range**	***P*-value**	**Median**	**Range**	***P*-value**
*Age*							
<66 years	26	17.6	1.8–81.3		231.1	51.7–620.1	
>66 years	25	15.9	1.6–46.2	0.510	292.3	21.1–619.8	0.763
							
*Laurén*							
Diffuse/ mixed	17	18.7	1.8–55.4		206.8	52.0–592.3	
Intestinal	34	16.6	1.6–81.3	0.460	284.7	21.1–620.1	0.238
							
*Differentiation*							
Well	21	15.5	1.8–55.4		214.2	52.0–620.0	
Moderate/ poor	28	17.5	1.6–81.3	0.824	295.5	21.1–620.1	0.303
							
*TNM*							
I	14	15.8	8.7–81.3		196.8	21.1–341.4	
II–IV	37	18.1	1.6–71.0	0.688	292.8	62.2–620.1	**0.010**
							
*Localization*							
Cardia	22	18.9	1.6–81.3		277.5	51.7–620.1	
Rest	29	13.9	1.8–46.2	**0.050**	214.2	21.1–619.8	0.555
							
*Diameter*							
<6 cm	30	16.6	3.2–46.2		205.7	21.1–534.8	
>6 cm	21	17.9	1.6–81.3	0.954	362.2	62.2–620.1	**0.004**
							
*Invasion*							
Subserosa	34	19.1	1.6–81.3		221.3	21.1–620.1	
Further	17	11.4	3.2–42.4	**0.034**	367.4	63.4–619.8	0.093
							
*Inflammation*							
Non/mild	43	15.9	1.6–55.4		274.6	21.1–619.8	
Severe	7	23.9	11.4–81.3	**0.010**	296.2	132.1–620.1	0.546
							
*Status* b							
Alive	17	13.9	1.8–46.2		209.5	21.1–385.0	
Deceased	34	18.8	1.6–81.3	0.208	286.3	51.7–620.1	0.215

TGF-*β*1=Transforming growth factor-*β1*; TNM, tumour node metastasis classification.

a Median and range in picogram per milligram protein.

b Tumour-associated death.

Bold *P*-values are considered significant.

**Table 2:** Uni- and multivariate Cox's proportional hazards analyses of total and active TGF-*β*1 levels in relation to tumour-associated survival of 51 gastric cancer patients

	**Univariate**			**Multivariate**		
**Parameter**	**HR**	**CI 95%**	***P*-value**	**HR**	**CI 95%**	***P*-value**
Age	1.258	0.637–2.484	0.509	1.584	0.662–2.716	0.240
Laurén classification	0.699	0.348–1.402	0.313	0.932	0.348–1.402	0.865
Differentiation	1.953	0.906–4.208	0.088	1.866	0.906–4.208	0.195
TNM classification	2.755	1.057–7.179	**0.038**	1.534	1.057–7.179	0.440
Tumour localization	2.379	1.092–5.180	**0.029**	1.916	1.092–5.180	0.151
Total TGF-*β*1	2.234	0.979–5.098	0.056	1.796	0.753–4.287	0.187
Active TGF-*β*1	2.339	1.065–5.138	**0.034**	2.125	0.934–4.836	0.072
High total/high active TGF-*β*1	3.108	1.240–7.788	**0.016**	2.763	1.061–7.199	**0.037**

Description of variables is shown in Table 1.

Bold *P*-values are considered significant.

**Table 3:** Spearman's correlations between the levels of total and active TGF-*β*1 and various proteinases and proteinase inhibitors in 51 gastric cancer homogenates

		**Active TGF-*β*1**		**Total TGF-*β*1**	
	**Assay**	***ρ***	***P*-value**	***ρ***	***P*-value**
uPA	ELISA	0.202	0.163	0.259	0.072
uPA	BIA	0.284	**0.048**	0.125	0.394
uPAR	ELISA	0.076	0.605	0.126	0.389
PAI-1	ELISA	0.195	0.185	0.198	0.176
PAI-2	ELISA	−0.181	0.219	−0.210	0.151
MMP-2	ELISA	0.219	0.149	0.296	**0.048**
MMP-2	BIA	0.253	0.111	−0.060	0.709
MMP-7	ELISA	−0.091	0.568	0.248	0.114
MMP-8	ELISA	0.717	0.240	0.111	0.449
MMP-9	ELISA	0.023	0.878	0.082	0.579
MMP-9	BIA	0.121	0.424	0.268	0.071
TIMP-1	ELISA	−0.039	0.821	0.045	0.794
TIMP-2	ELISA	−0.209	0.221	−0.064	0.711

PAI=plasminogen activator inhibitor; TIMP=tissue inhibitor of matrix metalloproteinase; uPA=urokinase plasminogen activator; uPAR=urokinase plasminogen activator receptor.

ELISA, total antigen level; BIA, activity level.

Bold *P*-values are considered significant.
